# Cardiorespiratory fitness level correlates inversely with excess post-exercise oxygen consumption after aerobic-type interval training

**DOI:** 10.1186/1756-0500-5-646

**Published:** 2012-11-21

**Authors:** Tomoaki Matsuo, Kazunori Ohkawara, Satoshi Seino, Nobutake Shimojo, Shin Yamada, Hiroshi Ohshima, Kiyoji Tanaka, Chiaki Mukai

**Affiliations:** 1 Space Biomedical Research Office, Japan Aerospace Exploration Agency (JAXA), 2-1-1, Sengen, Tsukuba, Ibaraki 305-8505, Japan; 2 Faculty of Informatics and Engineering, University of Electro-Communications, 1-5-1 Chofugaoka, Chofu, Tokyo, 182-8585, Japan; 3 Health Promotion and Exercise Program, National Institute of Health and Nutrition, 1-23-1, Toyama, Shinjuku, Tokyo, 162-8636, Japan; 4 Graduate School of Comprehensive Human Sciences, University of Tsukuba, 1-1-1 Tennodai, Tsukuba, Ibaraki, 305-8574, Japan

**Keywords:** Aerobic fitness, Maximal oxygen consumption, Cycling, Energy expenditure, Exercise physiology

## Abstract

**Background:**

The purpose of this study was to reveal any association between cardiorespiratory fitness level and excess post-exercise oxygen consumption (EPOC) using three cycling protocols with varying degrees of exercise intensity, i.e., sprint interval training (SIT), high-intensity interval aerobic training (HIAT), and continuous aerobic training (CAT).

**Findings:**

Ten healthy men, aged 20 to 31 years, attended a cross-over experiment and completed three exercise sessions: SIT consisting of 7 sets of 30-s cycling at 120% VO_2max_ with a 15-s rest between sets; HIAT consisting of 3 sets of 3-min cycling at 80~90% VO_2max_ with a 2-min active rest at 50% VO_2max_ between sets; and CAT consisting of 40 min of cycling at 60~65% VO_2max_. During each session, resting VO_2_, exercise VO_2_, and a 180-min post-exercise VO_2_ were measured. The net exercise VO_2_ during the SIT, HIAT, and CAT averaged 14.7 ± 1.5, 31.8 ± 4.1, and 71.1 ± 10.0 L, and the EPOCs averaged 6.8 ± 4.0, 4.5 ± 3.3, and 2.9 ± 2.8 L, respectively. The EPOC with SIT was greater than with CAT (*P* < 0.01) and HIAT (*P* = 0.12). Correlation coefficients obtained between subjects’ VO_2max_ and the ratio of EPOC to net exercise VO_2_ for SIT, HIAT, and CAT were −0.61 (*P* = 0.06), -0.79 (*P* < 0.01), and −0.42 (*P* = 0.23), respectively.

**Conclusions:**

Our data suggest that cardiorespiratory fitness level correlates negatively with the magnitude of EPOC, especially when performing aerobic-type interval training.

## Findings

### Introduction

A review paper regarding excess post-exercise oxygen consumption (EPOC) found that trained and conditioned individuals had a more rapid return from post-exercise metabolism to resting levels after exercise [1], whereas, another review paper found that training level had minimal influence on EPOC [2]. Thus, the association between fitness level and EPOC is still controversial, and both review papers [1,2] indicate further study should be conducted to confirm the association between fitness level and EPOC for more strenuous exercise bouts, such as levels greater than 80% maximal oxygen consumption (VO_2max_).

We have been developing two types of original exercise training protocols at the Japan Aerospace Exploration Agency (JAXA) which are better suited to astronauts during long-term spaceflight: sprint interval training (SIT) and high-intensity interval aerobic training (HIAT). In a previous study [3], we compared total energy expenditure (EE), including EPOC, induced by the two protocols with the total EE of traditional, continuous aerobic training (CAT). The study showed that exercise intensity had a noticeable effect on the EPOC. That is, although the SIT was the shortest duration among our three protocols, and the net exercise EE of the SIT (77 kcal) was only 20% of the EE of CAT (350 kcal) and 50% of the EE of HIAT (161 kcal), SIT’s EPOC during the 180-min post-exercise period was the greatest among the three protocols. In this short report, we examine associations between a subject’s cardiorespiratory fitness level and EPOC using our three exercise protocols.

## Methods

Detailed descriptions of the study protocol have been published elsewhere [3]. Briefly, our ten male subjects’ average values in age, height, body weight, body mass index and VO_2max_ were 24.0 ± 3.3 (range: 20–31) years, 170.8 ± 5.0 (162.1 - 175.6) cm, 61.9 ± 5.7 (54.6 - 69.7) kg, 21.2 ± 1.7 (18.4 - 23.3) kg·m^-2^, and 3.18 ± 0.45 (2.34 - 3.75) L·min^-1^ (or 52.0 ± 9.2 (36.2 - 61.4) ml·kg^-1^·min^-1^), respectively. The aim and design of this study were explained to every subject before each gave their written, informed consent. This study was conducted in accordance with the guidelines proposed in the Declaration of Helsinki. The Ethical Committee of both JAXA (reference number: 32-2-7) and University of Tsukuba (22–283) reviewed and approved the study protocol.

The subjects completed three experimental sessions separated by approximately 1 week. During each session, the subjects performed one of three exercise trials, i.e., SIT, HIAT, or CAT. The detailed protocols of the three cycling exercises are shown in Additional file 1: Table S1. All aspects of the experimental session remained the same during all three sessions except the actual exercise technique. The three sessions were implemented in random order.

On each session day, the subjects arrived at our laboratory at approximately 7:15 am. They were asked to minimize any physical activity (walking etc.) while en route from their home to the laboratory. Six subjects drove their own cars to the laboratory, and 4 subjects were picked up at their homes and driven to the building by a research assistant because they did not have their own cars. At 7:45 am, a subject would seat himself in a comfortable armchair and remain in a resting (seated) position without movement until 8:20 am. From 8:05 to 8:20 am (15 minutes), the subject was connected to a face mask of the indirect calorimeter for baseline data collection. Average VO_2_ data during the final 10 minutes were used as the baseline value. After the baseline measurements, we disconnected the face mask and allowed the subject to take a drink of water before riding on the cycling ergometer (75XL Ш, Konami, Tokyo, Japan) for the exercise phase. After the water, he was reconnected to the indirect calorimeter and started pedaling the ergometer at 8:25 am.

After the exercise phase, the subject returned to the resting position immediately but stayed connected to the indirect calorimeter for 10 minutes to measure immediate post-exercise VO_2_. After 10 minutes, we removed the face mask but the subject remained in the resting position for another 170 minutes. During the post-exercise phase, the subject remained in the resting position immobile. Ten minutes of VO_2_ measurements were started at 30, 60, 90, 120, 150, and 180 minutes post exercise. For each measurement session, average VO_2_ data during the final 5 minutes was used.

All gas exchange measurements were measured using the mixing chamber method with an open-circuit computerized indirect calorimeter (AE-310S, Minato Medical Science, Osaka, Japan). The ventilatory volume, VO_2_, and VCO_2_ were calculated every 15 seconds. The gas analyzer was calibrated before each trial. Coefficient of variation (CV) from the mean for the three resting VO_2_ measurements in the 10 subjects was 4.3%.

To measure each subject’s VO_2max_, we used the criteria described by Tanaka et al. [4] Briefly, after a 2-min warm up at 15 Watt (W), the subjects began the actual exercise protocol at a 30 W level. The workload was increased every minute by 15 W until volitional exhaustion. We determined the highest oxygen uptake achieved over 30 seconds as the VO_2max_.

To determine each subject’s exercise intensity during the exercise session, we used the VO_2max_ measurement data (i.e., values per minute for workload and VO_2_) and calculated a simple linear regression equation for each subject: Y = *β*x + c, with Y = workload (W), x = VO_2_ (ml), and ß and c as constants. Subsequently, percentage VO_2max_ data (ml) (e.g., 120% VO_2max_ for SIT) were applied to the equation, whereupon each subject’s exercise intensity (workload (W)) was determined.

To determine net exercise VO_2_ and EPOC, the area under the curve (AUC) for each phase was calculated for each subject using a statistical-software package (SAS version 9.2, SAS Institute Inc, Cary, NC, USA). As for the SIT and HIAT, periods of active rest were included in the calculation of the net exercise VO_2_.

To analyze differences among the three sessions, we used one-way analysis of variance (ANOVA), and applied Tukey-Kramer’s post hoc test when the difference was significant (*P* < 0.05) according to the results of ANOVA. We assessed the relationship between two measurement values with Pearson’s product moment correlation. We used SAS, version 9.2 (SAS Institute Inc, Cary, NC, USA) to analyze the data.

## Results

The net VO_2_ incurred with the HIAT (31.8 ± 4.1 L) was significantly lower than during the CAT (71.1 ± 10.0 L), but it was significantly greater than the net VO_2_ incurred during the SIT (14.7 ± 1.5 L). The EPOC was significantly greater with the SIT (6.8 ± 4.0 L) than with the CAT (2.9 ± 2.8 L), and it also tended to be greater than with the HIAT (4.5 ± 3.3 L) (*P* = 0.12). We observed no significant differences in the EPOC between HIAT and CAT.

Additional file 2: Figure S1 shows the relationship between subjects’ VO_2max_ and the ratios of EPOC to the net exercise VO_2_ during each exercise session. The ratio of EPOC to net exercise VO_2_ during the SIT (*r* = −0.61, *P* = 0.06) and the HIAT (*r* = −0.79, *P* < 0.01) correlated inversely with VO_2max_, whereas, we observed no significant correlation between these two variables for the CAT (*r* = −0.42, *P* = 0.23). As for the correlation between absolute EPOC value and VO_2max_, we observed a significant correlation for the HIAT (*r* = −0.67, *P* = 0.03), but no significant correlation for the SIT (*r* = −0.47, *P* = 0.17) and CAT (*r* = −0.34, *P* = 0.34).

## Discussion

Ohkawara et al. [5] showed a significant negative correlation between a subject’s fitness level and the EPOC under normal living conditions in a metabolic chamber. The significant correlation was observed not on a day with moderate-intensity physical activity but on a day with vigorous-intensity physical activity. Also, Singh et al. [6] recently showed that VO_2_ decline during the first minute of recovery after maximum exercise was faster in children with a higher VO_2max_. Our experimental results with a single bout of exercise were consistent with those studies’ results. That is, in the present study, while the ratio of EPOC to net exercise VO_2_ correlated inversely with VO_2max_, we observed this significant association when subjects performed a high-intensity interval exercise, especially an aerobic-type interval protocol (the HIAT), but not when they performed a moderate-intensity continuous protocol (the CAT). Although several studies [7-9] reported that training level did not significantly impact EPOC, the exercise intensities (30~70% VO_2max_) used in those previous studies were relatively low compared to our interval exercise protocols.

While the precise mechanism of EPOC remains unclear, physiological adaptation associated with improved fitness levels has been considered a factor potentially influencing EPOC [1]. For example, an enhanced lactate metabolism with elevated body temperature partly accounts for EPOC [2,10]. Trained individuals have better thermoregulatory capacities than untrained individuals because physical training enhances the sweating mechanism at a given level of the central sweating drive [11]. Therefore, elevated body temperature in untrained individuals could last longer than in trained individuals [12]. Moreover, subjects with lower VO_2max_ might produce more lactate than those with higher VO_2max_ especially during strenuous exercise. An enhanced lactate metabolism requires oxygen consumption for recovery. Thus, fitness level may contribute to the magnitude of EPOC.

In conclusion, we revealed that cardiorespiratory fitness level correlates inversely with the magnitude of EPOC, especially when performing an aerobic-type interval exercise.

## Competing interest

The authors declare no conflict of interest.

## Authors' contributions

The contributions of each author were as follows: TM and KO contributed to manuscript writing, developing study concept and design, data acquisition, and data analysis; SS contributed to manuscript revisions, data acquisition, and data analysis; NS contributed to manuscript revisions and data acquisition; SY and HO contributed to manuscript revisions and developing study concept and design; KT and CM represented the University of Tsukuba and JAXA, respectively, in this joint research project and contributed to manuscript revisions, developing study concept and design, and data acquisition. All authors read and approved the final manuscript.

## Supplementary Material

Additional file 1 **Table S1. **Three exercise protocols in this study. Click here for file

Additional file 2 **Figure S1. **Relationship between subject’s cardiorespiratory fitness level and excess post-exercise oxygen consumption for each exercise protocol: (a) SIT, (b) HIAT, (c) CAT. Correlation coefficients (*r*) between subjects’ maximal oxygen consumption (VO2max) (ml·kg-1·min-1) and the ratio of EPOC to net exercise VO2max (%) for SIT, HIAT, and CAT were −0.61 (*P* = 0.06), -0.79 (*P* < 0.01), and −0.42 (*P* = 0.23), respectively. SIT, sprint interval training; HIAT, high-intensity interval aerobic training; CAT, continuous aerobic training; EPOC, excess post-exercise oxygen consumption; VO2, oxygen consumption per minute. Click here for file
