# Supplementary material for: Cardiorespiratory fitness level correlates inversely with excess post-exercise oxygen consumption after aerobic-type interval training
Source: BMC Res Notes. 2012 Nov 21;5:646. doi: 10.1186/1756-0500-5-646 (PMC3527216; doi:10.1186/1756-0500-5-646)
Supplement: Additional file 1 — Table S1. Three exercise protocols in this study. [file 1756-0500-5-646-S1.pdf]

Table 1 Three exercise protocols in this study

	Program		Total time
Sprint Interval Training (SIT)	2 min (30 W, 60 rpm)<br>30 sec (120%VO_2max, over 85 rpm)<br>15 sec (rest)<br>3 min (30 W, 40 ~ 60 rpm)	Repeat 7 times (no rest at 7th)	10 min
High-intensity Interval Aerobic Training (HIAT)	2 min (30 W, 60 rpm)<br>3 min (85～90%VO_2max, 70 ~ 80 rpm)<br>2 min (50%VO_2max, 60 rpm)<br>3 min (85～90%VO_2max, 70 ~ 80 rpm)<br>2 min (50%VO_2max, 60 rpm)<br>3 min (80～85%VO_2max, 70 ~ 80 rpm)<br>3 min (30 W, 40 ~ 60 rpm)		18 min
Continuous Aerobic Training (CAT)	2 min (30 W, 60 rpm)<br>40 min (60～65%VO_2max, 60 rpm)<br>3 min (30 W, 40 ~ 60 rpm)		45 min
