# Supplementary figures and images for: Cardiorespiratory fitness level correlates inversely with excess post-exercise oxygen consumption after aerobic-type interval training
Source: BMC Res Notes. 2012 Nov 21;5:646. doi: 10.1186/1756-0500-5-646 (PMC3527216; doi:10.1186/1756-0500-5-646)

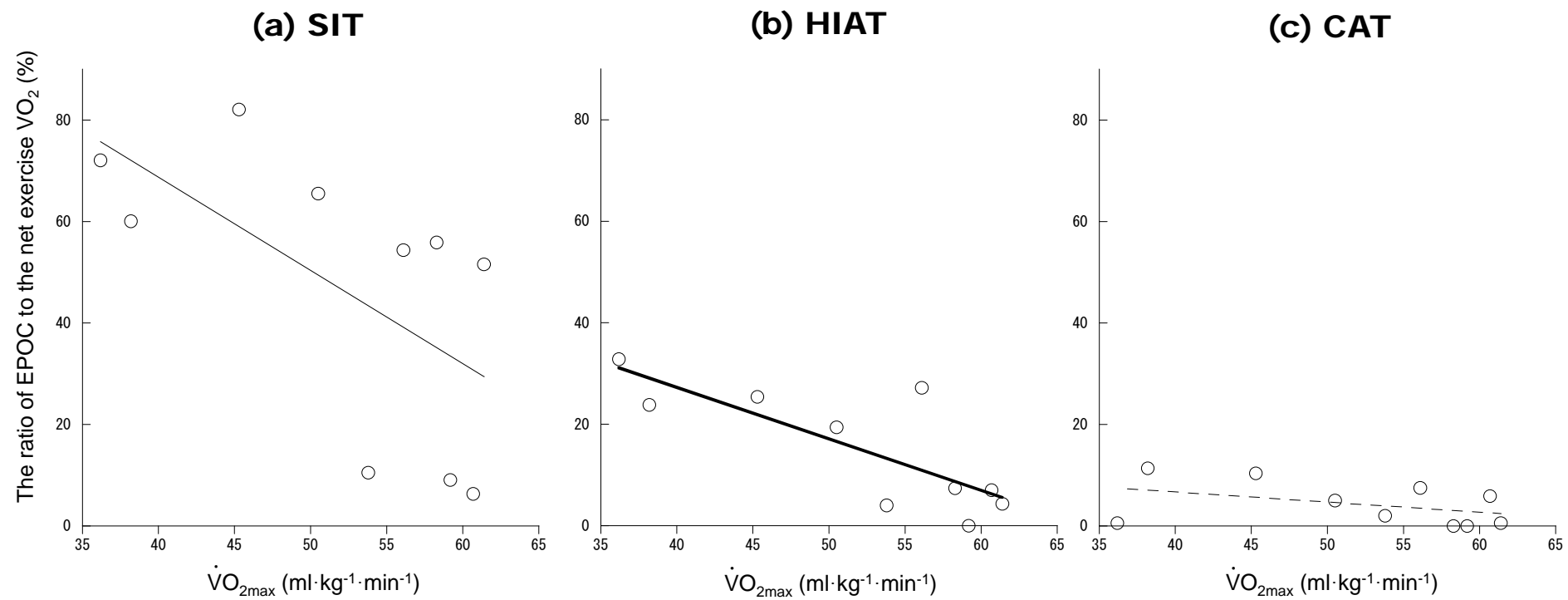

*Fig. 1*  
*T. Matsuo et al.*

Supplement: Additional file 2 — Figure S1. Relationship between subject’s cardiorespiratory fitness level and excess post-exercise oxygen consumption for each exercise protocol: (a) SIT, (b) HIAT, (c) CAT. Correlation coefficients (r) between subjects’ maximal oxygen consumption (VO2max) (ml·kg-1·min-1) and the ratio of EPOC to net exercise VO2max (%) for SIT, HIAT, and CAT were −0.61 (P = 0.06), -0.79 (P < 0.01), and −0.42 (P = 0.23), respectively. SIT, sprint interval training; HIAT, high-intensity interval aerobic training; CAT, continuous aerobic training; EPOC, excess post-exercise oxygen consumption; VO2, oxygen consumption per minute. [file 1756-0500-5-646-S2.pdf]
